# High-throughput cis-regulatory element discovery in the vector mosquito *Aedes aegypti*

**DOI:** 10.1186/s12864-016-2468-x

**Published:** 2016-05-10

**Authors:** Susanta K. Behura, Joseph Sarro, Ping Li, Keshava Mysore, David W. Severson, Scott J. Emrich, Molly Duman-Scheel

**Affiliations:** Eck Institute for Global Health, University of Notre Dame, Notre Dame, IN 46556 USA; Department of Biological Sciences, University of Notre Dame, Notre Dame, IN 46556 USA; Department of Medical and Molecular Genetics, Indiana University School of Medicine, 1234 Notre Dame Ave., South Bend, IN 46617 USA; Department of Computer Science and Engineering, University of Notre Dame, Notre Dame, IN 46556 USA

**Keywords:** Genome, FAIRE-seq, Dengue virus, Zika, Next generation sequencing, *Drosophila*

## Abstract

**Background:**

Despite substantial progress in mosquito genomic and genetic research, few cis-regulatory elements (CREs), DNA sequences that control gene expression, have been identified in mosquitoes or other non-model insects. Formaldehyde-assisted isolation of regulatory elements paired with DNA sequencing, FAIRE-seq, is emerging as a powerful new high-throughput tool for global CRE discovery. FAIRE results in the preferential recovery of open chromatin DNA fragments that are not bound by nucleosomes, an evolutionarily conserved indicator of regulatory activity, which are then sequenced. Despite the power of the approach, FAIRE-seq has not yet been applied to the study of non-model insects. In this investigation, we utilized FAIRE-seq to profile open chromatin and identify likely regulatory elements throughout the genome of the human disease vector mosquito *Aedes aegypti*. We then assessed genetic variation in the regulatory elements of dengue virus susceptible (Moyo-S) and refractory (Moyo-R) mosquito strains.

**Results:**

Analysis of sequence data obtained through next generation sequencing of FAIRE DNA isolated from *A. aegypti* embryos revealed >121,000 FAIRE peaks (FPs), many of which clustered in the 1 kb 5’ upstream flanking regions of genes known to be expressed at this stage. As expected, known transcription factor consensus binding sites were enriched in the FPs, and of these FoxA1, Hunchback, Gfi, Klf4, MYB/ph3 and Sox9 are most predominant. All of the elements tested in vivo were confirmed to drive gene expression in transgenic *Drosophila* reporter assays. Of the >13,000 single nucleotide polymorphisms (SNPs) recently identified in dengue virus-susceptible and refractory mosquito strains, 3365 were found to map to FPs.

**Conclusion:**

FAIRE-seq analysis of open chromatin in *A. aegypti* permitted genome-wide discovery of CREs. The results of this investigation indicate that FAIRE-seq is a powerful tool for identification of regulatory DNA in the genomes of non-model organisms, including human disease vector mosquitoes.

**Electronic supplementary material:**

The online version of this article (doi:10.1186/s12864-016-2468-x) contains supplementary material, which is available to authorized users.

## Background

Vector mosquitoes inflict more human suffering than any other organism and spread diseases that kill more than one million people each year. Mosquito-borne illnesses, among the most complex infectious diseases to prevent and control, have resurged worldwide and pose threats for epidemic outbreaks in developed countries, including the United States. Given poor progress in vaccine development and distribution, mosquito control is the primary mechanism for disease control. However, the emergence of insecticide resistance and a lack of support for mosquito control programs compromise strategies for managing mosquitoes, resulting in a need for the development of new approaches to combat these insect vectors of human disease [1]. The mosquito genome projects have facilitated research in many new facets of mosquito biology [2–5]. However, despite substantial progress in mosquito genomic and genetic research, very few cis-regulatory elements (CREs), DNA sequences that control gene expression, have been identified in the mosquito genomes. CRE discovery is challenging because these elements are typically short sequences contained within vast stretches of intergenic DNA [6]. Although computational approaches resulted in the identification of a number of mosquito CREs [6], CRE discovery has not continued to progress, and regulatory regions for most mosquito genes remain unknown. This deficiency—a significant gap in our basic knowledge of mosquito genetics—has resulted in a lack of drivers to manipulate gene expression in selected tissues at specific times, the inability to properly dissect gene regulatory networks in mosquitoes, and difficulty in understanding the biological meaning of genetic variation that resides in non-coding regions.

FAIRE-seq, formaldehyde-assisted isolation of regulatory elements paired with DNA sequencing [7], is emerging as a powerful new approach for global biochemical isolation of CREs through their lack of association with nucleosome proteins. FAIRE, which exploits the ability of formaldehyde exposure to form crosslinks between interacting nucleosomal histones and DNA, has emerged as a powerful new approach for genome-wide identification of regulatory elements [7–9]. During the FAIRE process, chromatin is cross-linked with formaldehyde, sheared, and then phenol-chloroform extracted, permitting recovery of open chromatin DNA fragments that are not bound by nucleosomes, an evolutionarily conserved indicator of regulatory activity [10]. FAIRE has many advantages over alternative methods, one being that the recent pairing of this technique with next-generation sequencing, FAIRE-seq, permits straightforward and genome-wide high throughput detection of CREs [7]. FAIRE-seq is technically straightforward and involves fewer steps, reagents, and variables than alternative methodologies. Use of FAIRE eliminates the tedious titration of DNAse activity and limits concerns about variations in nuclei preparations that affect other protocols. Unlike chromatin immunoprecipitation, there is no dependence on antibody supply/quality or the production of tagged proteins. Also, unlike DNAse sensitivity assays, FAIRE requires no cellular treatments prior to crosslinking and better captures the endogenous chromatin state [8].

Here, we describe a FAIRE-seq investigation in the human disease vector mosquito *Aedes aegypti. A. aegypti,* a container-breeding mosquito that is closely associated with humans and their urban dwellings, transmits viruses that cause Zika, yellow fever, chikungunya, and dengue, the most widespread and significant arboviral disease in the world. A third of the world’s population is at risk for dengue virus (DENV) infection, a leading cause of illness and death in the tropics and subtropics. DENV is transmitted to as many as 400 million people each year when they are bitten by infected *Aedes* mosquitoes [1]. *A. aegypti* strains that are susceptible (Moyo-S) and refractory (Moyo-R) to DENV infection have been selected from a common genetic background [11, 12]. Genome-wide transcriptome comparisons suggest that modular expression of genes is significantly different in the two strains and that many genes (~2500) may contribute to susceptible/refractory responses of *A. aegypti* to DENV infection [13]. In a companion study, next generation sequencing identified >13,000 single nucleotide polymorphisms (SNPs) in the two strains [14–16]. In this investigation, FAIRE-seq analysis of open chromatin in *A. aegypti* permitted genome-wide discovery of cis-regulatory elements, which facilitated analysis of genetic variation in non-coding cis-regulatory elements that may contribute to mosquito susceptibility and responses to DENV infection.

## Methods

### Ethics

This study did not include human subjects, human data, or vertebrate animals.

#### Mosquito rearing and egg collection

The *A. aegypti* Liverpool-IB12 (LVP-IB12) strain, from which the genome sequence [3] was derived, was used in this investigation. Mosquitoes were maintained in an insectary at 26 °C, ~80 % humidity, under a 12 h light and 12 h dark cycle with 1 h crepuscular periods at the beginning and end of each light cycle. Larvae fed on a suspension of dried beef liver powder, and adults were provided cotton soaked with 10 % sugar solution. For blood feeding adult females, an artificial membrane feeding system was used in conjunction with sheep blood purchased from Hemo-Stat Laboratories (Dixon, CA). Females deposited eggs on paper toweling during two-hour eggs collections. Eggs were maintained in the insectary for an additional 49 h.

#### FAIRE

DNA was prepared using a modified version of the *Drosophila melanogaster* embryonic tissue protocol [17], which is based on Simon et al. [7] methodology. DNA processed in this manner will hereafter be referred to as FAIRE DNA. In summary, 100 mg of eggs were treated with 50 % bleach for 3 min. and then rinsed thoroughly with distilled water. Crosslinking was performed for 15 min using 10 mL 0.4 % formaldehyde in PEM buffer (100 mM PIPES disodium salt, 2 mM EGTA, 1 mM magnesium sulfate; pH 7) in a 60 °C water bath. Following crosslinking, which was quenched through addition of glycine, embryonic nuclei were pelleted through centrifugation at 1500 RCF at 4 °C for 2 min. The nuclei were resuspended in Buffer A (10 mM HEPES pH 8, 10 mM EDTA, 0.5 mM EGTA, 0.25 % Triton X-100) and homogenized with a pestle. The lysate was passed through Miracloth to remove debris. The nuclei were pelleted again, resuspended in FAIRE lysis buffer (2 % Triton X-100, 1 % SDS, 100 mM NaCl, 10 mM Tris-Cl pH 8, 1 mM EDTA), and subjected to six rounds of bead beating with 0.5 mm glass beads/vortexing at 4 °C. Chromatin was sonicated using a Branson 250 ultrasonifier outfitted with a microtip (6 × 30 s with 1 s pulse, 0.5 s stop, 22 % amplitude), generating a size range of 300–500 bp fragments. Soluble chromatin was then recovered following centrifugation at 15,000 G for 5 min at 4 °C. FAIRE DNA was recovered from the cell lysate through phenol chloroform extraction in which open-chromatin DNA was recovered from the aqueous phase. The recovered open chromatin was treated with RNAse A and proteinase K, and then purified through a Qiaquick spin-column (Qiagen, Valencia, CA). The yield of FAIRE DNA (~1 μg) was sufficient for sequencing and corresponded to ~1 % of total DNA, well within the acceptable yield recommended by Simon et al. [7].

#### Illumina library construction and sequencing

FAIRE DNA Illumina libraries were prepared by the University of Notre Dame Genomics and Bioinformatics Core facility using the TruSeq kit (Illumina, San Diego, CA) per the manufacturer’s guidelines. 1 μg of FAIRE-enriched DNA was processed following the Illumina TruSeq DNA LT sample preparation protocol. Briefly, samples were end repaired and 3’ adenylated. Illumina adapters were ligated to the template, then purified and size-selected (for 100–300 bp fragments) on an agarose gel. A 10-cycle PCR reaction enriched for ligation products containing both Illumina adapters. Sample concentration was measured on a Qubit fluorometer (Life Technologies, Grand Island, NY), and sample size distribution was assayed on an Agilent 2100 Bioanalyzer (Agilent Technologies, Inc., Santa Clara, CA). Next-generation Illumina Sequencing (HiSeq 50 bp paired-end sequencing) with an Illumina HiSeq 2000 (BGI Americas Corporation, Cambridge, MA) was used to generate ~150 million reads for each of three replicate FAIRE DNA preparations.

#### Analysis of FAIRE sequencing data

Raw sequences were trimmed of adapters with Trimmomatic [18] version 0.32 and assessed for quality with FastQC [19] version v0.10.1. Trimmed sequences were aligned to version three of the *A. aegypti* scaffolds reference genome [20, 21] using the backtrack algorithm in BWA [22] version 0.5.9-r16 for three replicates. The third replicate, however, required further processing due to an unknown segmentation fault. A subset of fifty thousand reads containing one or more reads that could not be processed by BWA was located using Makeflow_BWA [23] and removed to enable successful alignment. Reads were further filtered with Samtools [22] before checking for reproducibility of replicates with the Irreproducible Discovery Rate (IDR) framework as described [24, 25] and calling peaks with MACS2 [26]. These peaks are hereafter referred to as FAIRE peaks (FPs). The maximum insert size itself was estimated empirically using BWA and resulted in calling peaks with an extent size of 550 bp.

#### Prediction of known transcription factor binding sites

Overrepresentation analysis of FPs for known transcription factor binding sites (JASPAR eukaryote, data downloaded in March 2014) was performed. The ‘Clover’ (Cis-eLement OVERrepresentation) tool [27] was used to determine motif incidences in the FPs. In the first step, the degree of motif [transcription factor (TF)–binding] incidences in the FPs was expressed by a score that was calculated. In the second step, the *p*‐value was obtained from the raw score, which is essentially the probability that the motif incidence score is either equal or greater to that of the threshold (user defined, set to a default value of 6) by chance. The background sequences were generated for Clover analysis as described previously [28].

#### Association of FPs with gene expression data in embryos

To determine if FP sequences identified in this study are associated with immediate (<1 kb) 5’ upstream regulatory regions of genes expressed during this embryonic stage, FPs were compared with available 48–52 h embryonic transcriptome data previously described by Akbari et al. [29].

#### Identification of SNPs in FPs

Single nucleotide polymorphisms (SNPs) within FP sequences were determined from a set of SNP data generated from a companion study (which will be described in its entirety elsewhere) in which Illumina sequencing of genomic DNA from two *A. aegypti* lab strains, Moyo-S and Moyo-R, was performed [14–16]. Both strains have been described previously [11, 12]. The SNP positions predicted within each supercontig were used to assess if they are present within the FP start and end coordinates within the same supercontigs.

#### Analysis of genomic distribution of FPs

The map locations of FPs were compared relative to the genomic coordinates of gene models (both protein coding and non-coding genes) to determine their associations. All 3’ and 5’ untranslated region (UTR) sequences of *A. aegypti* genes were obtained from VectorBase (VB) [20, 21], and the UTR regulatory elements present in those sequences were identified by using UTRScan [30].

#### Transgenic reporter generation and analysis

FP DNA sequences of interest were PCR-amplified from *A. aegypti* genomic DNA and cloned into plasmid *pattBnucGFPs*, a ϕC31-enabled *Drosophila* transformation vector containing enhanced green fluorescent protein (EGFP) under the control of a minimal *hsp70* promoter (graciously provided by M.S. Halfon). Transgenic *Drosophila* were produced at Rainbow Transgenic Flies, Inc. (Camarillo, CA) by injection into line *PBac{y[+]-attP-9A}VK00027* (Bloomington stock 9744 [31]). Tissue from transgenic animals was collected and fixed as described previously [32]. Tissues were mounted and imaged on a Zeiss 710 confocal microscope using Zen software or a Zeiss Axioimager equipped with a Spot Flex camera and Spot Digital Imaging software. Images were processed with Adobe Photoshop software.

## Results and discussion

### Gene-centric distribution of FPs

**Fig. 1:**
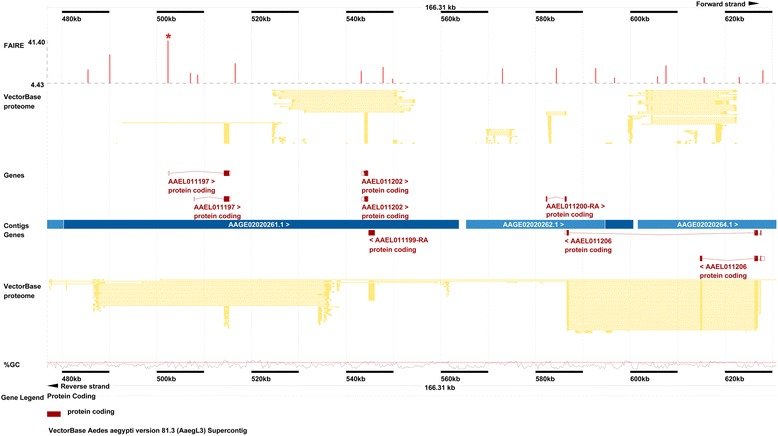
Visualization of FAIRE-seq data in the VB genome browser. FAIRE-seq reads [36] mapped to version three of the *A. aegypti* scaffolds reference v.4 [20, 21] are accessible through the VB Genome Browser. FPs in the supercontig 1.551: 476,900–630,100 bp region are shown. The peak marked with an asterisk corresponds to reporter A in Table 2. The gene accession numbers noted here and in all subsequent tables and supplementary materials correspond to VB [20, 21]

FAIRE-seq open chromatin profiling was performed to identify regulatory DNA in the *A. aegypti* genome. For these studies, embryos were collected 50 +/− 1 h after eggs were laid, a time point that coincides with the onset of axon pathfinding [33], a topic of interest to our laboratory [33–35], and a time period for which transcriptome data are available [29]. The FAIRE procedure was optimized using these *A. aegypti* embryos as input tissue until a 1 % FAIRE DNA yield (with respect to the total input DNA) was obtained, an amount that is within the optimal range noted by Simon et al. [7]. FAIRE-seq replicate experiments were highly reproducible, as evidenced by IDR [24, 25] analysis (Additional file 1) which detected no significant differences between three replicate experiments, the data from which were subsequently combined for downstream analyses. FAIRE-seq reads [36] were mapped to version three of the *A. aegypti* scaffolds reference v.4 [20, 21] and have been made accessible through the VB Genome Browser (Fig. 1). In total, FAIRE-seq open chromatin profiling resulted in the identification of ~121,600 FPs (Additional file 2) [36], a number which is consistent with results reported in other systems [7].

### FAIRE-seq identifies regulatory elements in the *A. aegypti* genome

**Fig. 2:**
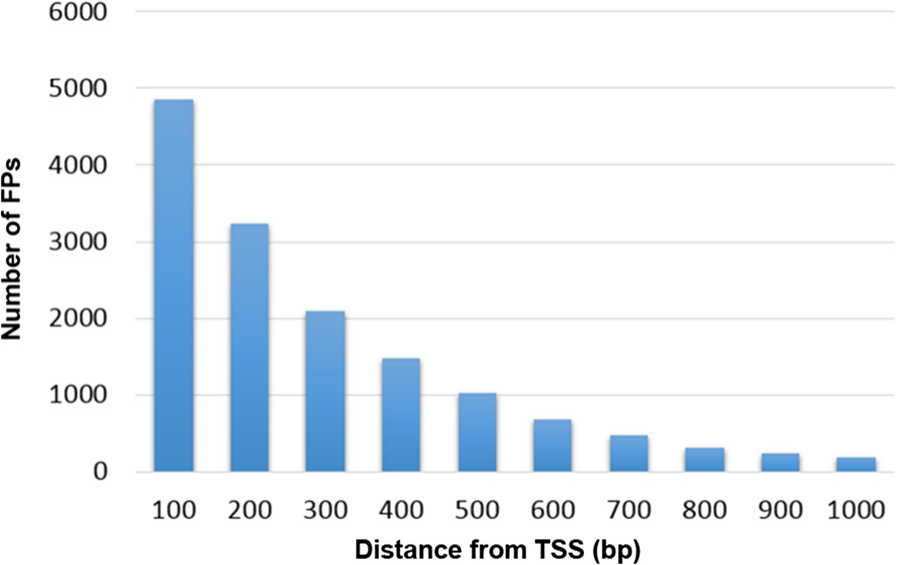
FPs are enriched around TSSs. The aggregated FAIRE signal adjacent to transcription start sites (TSSs) was increased within 100–200 bp upstream of TSSs

**Fig. 3:**
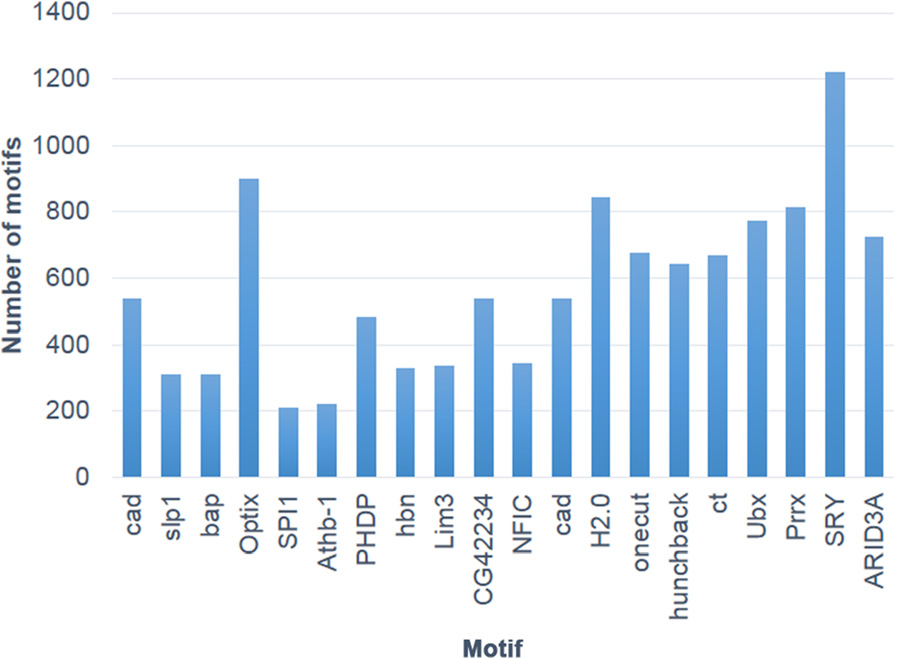
Top TF-binding sites significantly enriched in FPs. The top 20 TF-binding sites that are significantly enriched in the FPs are shown

Several lines of evidence indicate that FAIRE reliably identified regulatory elements in *A. aegypti*. In total, 5 % of the FPs were found to be located at proximal promoters (Additional file 3), a figure that is consistent with reports in other systems [37, 38]. These sequences can include both core promoters as well as regulatory elements adjacent to the promoters. However, one limitation of FAIRE is that other assays, including DNAse-seq, may be better for identification of nucleosome-depleted promoters of highly expressed genes [7, 38]. The aggregated FAIRE signal around all transcription start sites (TSSs) showed higher frequency within 100–200 bp upstream of TSSs (Fig. 2), which is also expected [7]. Furthermore, transcription factor binding sites are often associated with regulatory sequences in eukaryotes [39]. Known transcription factor binding sites are enriched in the FPs (Table 1) with respect to the rest of the genome. Among them, the top 20 TF-binding sites that are significantly enriched in FPs are shown in Fig. 3. Of these, the following consensus sequences are most abundant: FoxA1, Gfi, Hunchback, Klf4, MYB/ph3 and Sox9. Some of these elements, particularly Hunchback and FoxA1, were also abundant in FPs localized to the UTRs and intragenic regions (these regions are discussed further below).

**Table 1:** Consensus binding sites enriched in FPs

TF binding	Motif incidences
CG11617	2813
FOXA1	1225
SRY	1222
Optix	901
H2.0	845
Prrx	813
Ubx	773
ARID3A	723
Onecut	678
ct	670
Hunchback	641
cad	538
CG42234	538
PHDP	481
NFIC	345
Lim3	338
hbn	330
bap	312
slp1	312
Athb-1	222
SPI1	210
CG4328	204
E5	194
Lhx3	165
CG34031	160
Dr	160
Dll	155
HIF1A:ARNT	135
CF2-II	133
Vsx2	109
zen2	105

Known transcription factor binding sites were found to be enriched in FPs (with respect to the entire *A. aegypti* genome). Motif incidences in FPs (>100) as determined by Clover are shown here. In each case, Clover shows a significant *p* value for enrichment of the motif in FPs with the genome sequence used as background sequences for comparison

Next, the proximity of FPs with respect to genes known to be expressed in *A. aegypti* embryos was examined. The developmental transcriptome o*f A. aegypti* was recently described [29]. RNA-seq data is available for 48–52 h embryos [29], which includes the time span analyzed in the FAIRE-seq study (49–51 h). Analysis of the FAIRE data with respect to RNA-seq data revealed that 78 % (4733) of genes expressed at this developmental stage have a 5’ flanking (within 1 kb upstream of the TSS) or intragenic FP. In the majority of cases (89 %), these FPs are located within 1 kb upstream of the TSS of the gene, and in the other 11 % of cases, they reside within the genes in question. For cases in which FPs localized to within 100 bp upstream of the TSS, the gene has a relatively higher expression level (1.4 fold) compared to genes for which FPs were localized to more distant locations. This suggests that positional proximity of regulatory sequences may influence expression of the downstream genes, at least during embryonic development in *A. aegypti*.

**Fig. 4:**
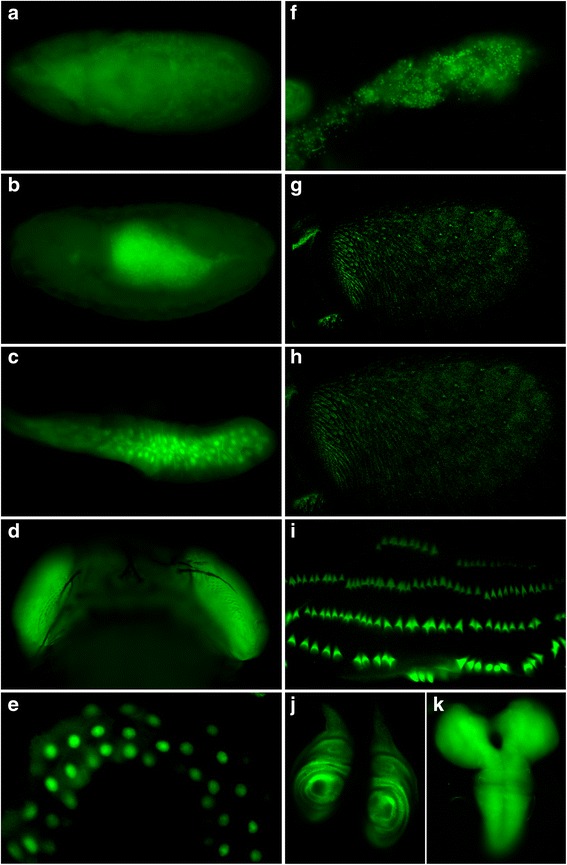
FP DNA sequences promote gene expression in vivo*.* GFP reporter expression (from the reporters indicated in Table 2) was detected in the following *D. melanogaster* tissues: embryo (reporter A in **a**; whole-mount is shown), embryonic midgut (reporter B in **b**), third instar larval salivary gland (reporter C in **c**), pupal eyes (reporter D in **d**), third instar larval gut (reporter E in **e**), third instar larval fat body (reporter F in **f**), adult antenna (reporter G in **g**, reporter H in **h**), third instar larval denticle belts (reporter I in **i**), third instar larval leg discs (reporter J in **j**), and the third instar larval brain (reporter K in **k**)

To further assess whether FAIRE-seq identified regulatory regions in the *A. aegypti* genome, a number of FP DNA sequences were tested for their ability to drive gene expression in vivo*.* Although transgenic technology is available in *A. aegypti*, transgenic generation in *D. melanogaster* is mature, quick, and inexpensive, and it is relatively easier and cheaper to maintain fruit fly stocks. Given the merits of *Drosophila* transgenics, 11 *A. aegypti* FP DNA sequences (Table 2) were cloned into a *Drosophila* transformation vector containing EGFP under the control of a minimal *hsp70* promoter. 100 % of these elements were confirmed to drive EGFP reporter expression in *D. melanogaster* (Fig. 4). Although the FAIRE-seq study was performed in *A. aegypti* embryos, in addition to embryos (Fig. 4a, b), a number of the FP sequences identified were found to be capable of driving gene expression at later stages of the *Drosophila* life cycle, including the larval (Fig. 4c, e, f, i, j, k), pupal (Fig. 4d), and adult (Fig. 4g, h) stages. These findings suggest that some of the regulatory sequences that function in embryos are also active at later stages of the life cycle, including adults. However, a recent FAIRE-seq study in *D. melanogaster* showed that following cell-type specification in the appendages, open chromatin profiles changed as the appendages progressed toward terminal differentiation, suggesting that stage-specific functions also require opening of new regulatory sites or the closing of existing sites [17]. In the future, it will be interesting to generate *A. aegypti* transgenic reporters to examine the activity of the regulatory elements assessed here in *Drosophila* (Table 2, Fig. 4) at multiple stages of the mosquito life cycle.

**Table 2:** FAIRE transgenic reporters

Reporter	FP	Enrichment *p*-value	Flanks VB Gene #	Gene TSS
A	Supercont1.551:501192–503018	8.27104E-39	AAEL011197	Supercont1.551: 507860
B	Supercont1.440:550819–551917	2.40519E-06	AAEL009947	Supercont1.440: 551773
C	Supercont1.381:720103–720682	0.001345953	AAEL009224	Supercont1.381: 720570
D	Supercont1.2641:1068–1902	1.61139E-10	AAEL015489	Supercont1.2641: 1778
E	Supercont1.911:297903–298590	3.79289E-08	AAEL013757	Supercont1.911: 294736
F	Supercont1.16:273854–274851	7.14776E-11	AAEL000765	Supercont1.16: 283224
G	Supercont1.237:1279560–1280173	0.000437431	AAEL007110	Supercont1.237: 1269860
H	Supercont1.174:341062–341799	3.97961E-10	AAEL005776	Supercont1.174: 357279
I	Supercont1.128:2089446–2090042	0.000177296	AAEL004719	Supercont1.128: 2098660
J	Supercont1.635:654750–655775	6.27856E-26	AAEL011943	Supercont1.635: 655775
K	Supercont1.160:604315–605761	1.43933E-45	AAEL005507	Supercont1.160: 435622

The FP DNA sequences listed were tested for their ability to drive GFP reporter expression in *D. melanogaster* transgenics. These reporter assays confirmed that all of the indicated sequences function as regulatory elements (see Fig. 4 for results). The *p*-values for enrichment, flanking genes, and TSSs of the flanking genes are noted

This investigation identified regulatory elements that drive gene expression in a wide variety of *D. melanogaster* tissues, including the eye (Fig. 4d), gut (Fig. 4b, e), salivary gland (Fig. 4c), fat body (Fig. 4f), olfactory system (Fig. 4g, h), denticle belts (Fig. 4i), leg imaginal discs (Fig. 4j), and brain (Fig. 4k). These studies in transgenic fruit flies provide functional validation of the FAIRE-seq data, suggesting that this investigation has succeeded in the goal of identifying regulatory elements. The *Drosophila* reporter assays permitted high throughput analysis of *A. aegypti* FAIRE-seq data in a manner which is not presently possible in mosquitoes, but which will lay the groundwork for full characterization of select CREs of interest directly in *A. aegypti* in future studies*.* To this end, we are presently pursuing a screen in *Drosophila* that aims to identify FP sequences that promote gene expression in tissues of vector importance (i.e., the olfactory system, midgut, salivary gland, and fat bodies). The elements selected for transgenic reporter studies presented here (Table 2, Fig. 4) were chosen based on their potential for driving gene expression in these tissues, which was estimated largely through examination of available *Drosophila* or mosquito gene expression data. Following the *Drosophila* screen, a number of regulatory elements that are confirmed to promote gene expression in tissues of interest will be tested directly in *A. aegypti* transgenics. It will be interesting to determine if these drivers promote comparable gene expression patterns in both *D. melanogaster* and *A. aegypti.* It is anticipated that if *A. aegypti* CREs drive tissue-specific reporter expression in *Drosophila* that mimics the activity of these elements in *A. aegypti,* the CREs are likely to function similarly in other dipterans, including additional vector mosquito species that are more closely related to *A. aegypti* than are fruit flies.

### FPs mapping to UTRs

**Fig. 5:**
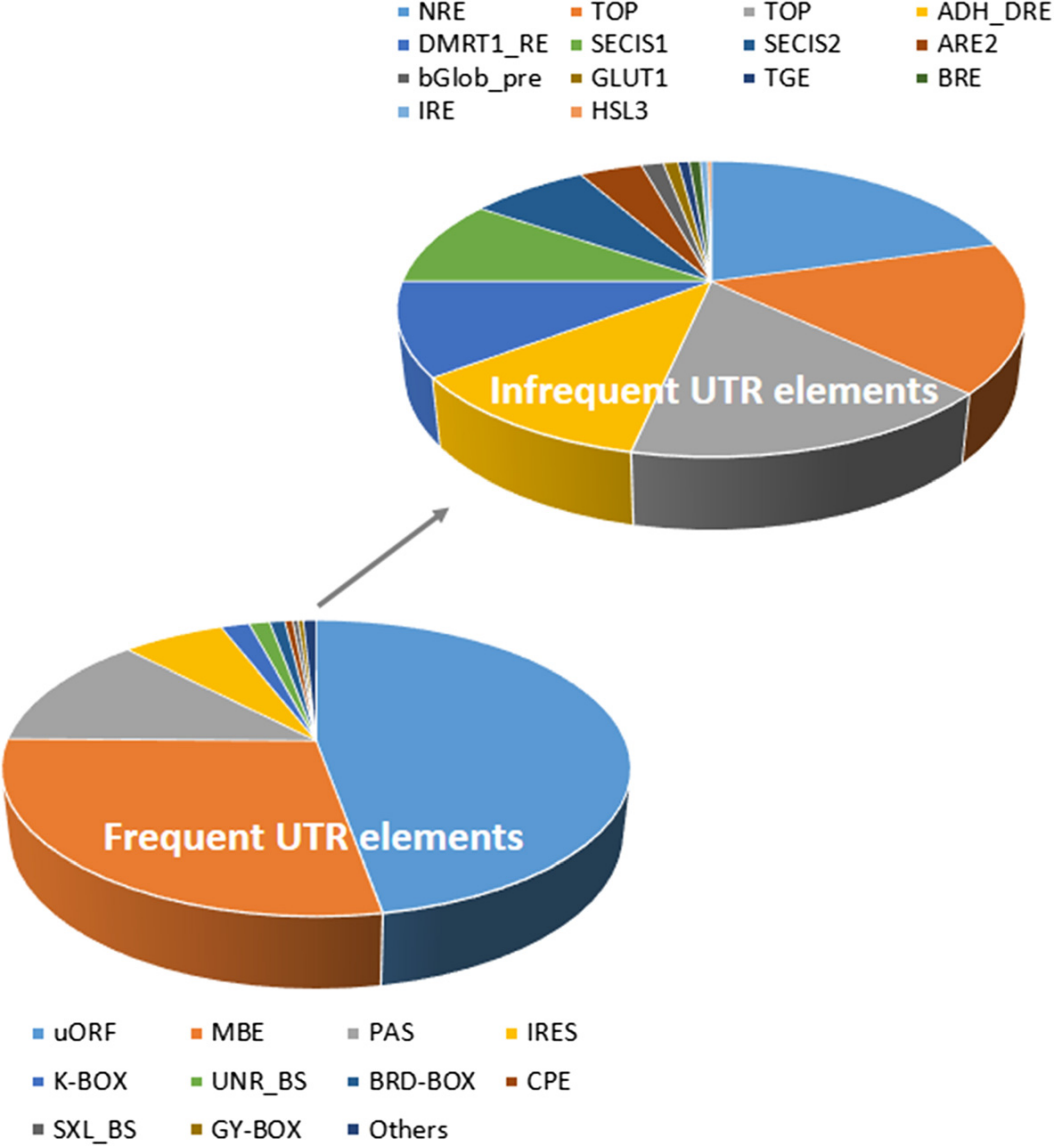
Known regulatory elements in UTR-linked FPs. Lower pie chart: uORF, MBE, PAS, and IRES motifs are the most frequent regulatory sequences observed in UTR-linked FP sequences. Upper pie chart: Infrequently observed UTR elements contained in the “Others” category of the lower pie chart are shown

The 5’ UTR, the mRNA leader sequence directly upstream of the translation initiation codon, plays critical roles in the regulation of transcription as well as translation in eukaryotes [40]. The regulatory roles of 3’ UTRs are also well documented [41]. Using the UTR-Scan tool [30], a total of 405 and 764 FPs were identified in the 5’- (Additional file 4) and 3’- (Additional file 5) UTRs of genes, respectively. Interestingly, UTR-linked FPs appear to have a biased composition of known regulatory elements. Sequences representing the upstream open reading frame (uORF), Musashi-binding element (MBE), polyadenylation signal (PAS), and internal ribosome entry site (IRES) represented the most frequent regulators based on rank order analysis of their frequencies (Fig. 5). Interestingly, approximately half of all the UTR regulatory elements identified by UTRScan represented uORF elements. These elements, which are found in the 5' region of a mRNA transcript, are capable of regulating protein production and impact organismal development and growth in fungi, plants, and animals, including insects [42].

### Intragenic regulators

Although intragenic regulators are significant in mammalian genomes [43, 44], such regulatory elements remain poorly understood in arthropods. However, a recent genome-wide study in *D. melanogaster* has revealed that many enhancers are localized within genes [45]. To know if FPs in *A.aegypti* coincide with intragenic enhancers, FPs localized within genes (Additional file 6) were analyzed. 767 FPs were identified in the exons of 744 genes (Additional file 7), and a total of 4434 identified FPs were localized within the intron sequences of 3747 genes (Additional file 8). 3991 FPs were identified in the exon-intron boundaries of 3592 genes (Additional file 9). The aggregated percentage of intragenic FPs is about 7.5 % of all the FPs identified in the genome. The relative distribution of FPs in upstream vs. intragenic sites is similar to the distribution bias of enhancer elements in *D. melanogaster* genes [45], indicating possible evolutionary conservation of intragenic regulation of genes in dipterans.

### Association of FPs with non-coding genes

**Fig. 6:**
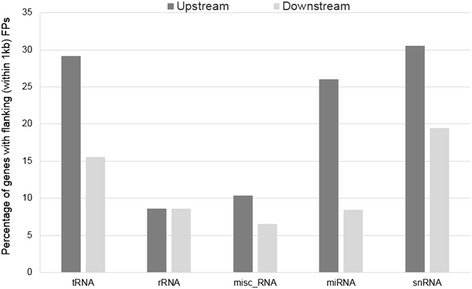
FPs upstream of non-coding *A. aegypti* genes. tRNA, rRNA, snRNA, and microRNAs have FPs within 1 kb upstream of their TSSs

Major classes of *A. aegypti* non-coding genes including tRNA, rRNA, snRNA, and microRNAs, also have FPs within 1 kb upstream of their TSSs (Fig. 6; Additional file 10). The precursor transcripts of miRNA genes are known to be regulated by cis-acting elements [46, 47], thus suggesting that FPs identified within 1 kb upstream of 43 *A. aegypti* miRNA genes may play a significant role in regulating synthesis of their precursors. Similarly, specific sequences upstream of tRNA genes are also known to regulate synthesis of tRNA molecules [48]. 287 FPs are associated with upstream sequences within 1 kb of ~30 % of *A. aegypti* tRNA genes, indicating possible cis-regulation of isoacceptor tRNAs. In addition to miRNA and tRNA, snRNA and rRNA genes have also been found to have association with cis-acting regulators [49, 50], and FPs were found to flank these genes in *A. aegypti.*

### Genetic variation in the regulatory regions of DENV susceptible and refractory mosquito strains

Identifying and understanding genetic variation in non-coding regions, which is often challenging even in genetic model organisms, is particularly difficult in non-models. In a recent study, Illumina sequencing of *A. aegypti* Moyo-S and Moyo-R genomic DNA revealed genome-wide genetic variation between the two strains [14–16]. In total, 13,158 high quality SNPs distributed throughout the genome (1321 supercontigs) were identified. These data were used to map the SNPs to FP sequences identified in the present study. The mapping data revealed that 3365 SNPs reside in FP DNA sequences (Additional file 11). To determine if there was any assortment bias of FAIRE-associated SNPs, bootstrap randomized sampling was performed as described earlier [51] in which 10,000 FPs were randomly sampled to determine how many contained a SNP. Performing a 100,000 randomized sampling, it was determined that on average, 28 SNPs were detectable in every 1000 FPs (Additional file 12). This pattern was consistent in each repeat of randomization, clearly suggesting no bias in the assortment of SNPs in the FPs.

Although several intensive studies have assessed SNPs in *A. aegypti* in a genome-wide manner [52–54], our studies are to our knowledge the first attempt to map *A. aegypti* SNPs to regulatory sequences across the genome. Likewise, this investigation is to our knowledge the first to map genetic polymorphism data from pathogen susceptible and refractory host strains to non-coding regulatory regions across the genome of the host. FAIRE-seq could be used for comparable analyses in malaria vector mosquitoes or hosts for other disease-causing pathogens. As discussed by Meyer and Thye [55], the next steps will be to identify causative genetic variation and the functionality of associated factors. To this end, SNPs in putative *A. aegypti* regulatory sequences [14–16] that flank genes with differential transcriptome profiles in Moyo-S vs. Moyo-R strains are presently being functionally characterized.

## Conclusions

The results of this investigation indicate that FAIRE-seq is a powerful tool for identification of regulatory DNA in the genomes of non-model or emerging model organisms, including human disease vector mosquitoes. In this study, FAIRE-seq analysis of open chromatin in *A. aegypti* embryos permitted genome-wide discovery of >121,000 regulatory elements throughout the genome of *A. aegypti.* Many of these sequences clustered in the 1 kb 5’ upstream flanking regions of genes known to be expressed at this stage. Known transcription factor consensus binding sites were enriched in the FPs, and all of the elements tested in vivo were confirmed to drive reporter gene expression in assays conducted in *D. melanogaster* transgenic animals. Of the >13,000 single nucleotide polymorphisms (SNPs) recently identified in DENV-susceptible and refractory mosquito strains, over one-quarter mapped to FPs, suggesting that genetic variation in regulatory sequences may contribute to the susceptibility/refractoriness of *A. aegypti* strains to DENV infection.

### Supporting data

Data sets supporting the results of this article are available within the article and its additional files, in the Sequence Read Archive (SRA) repository [56] (accession numbers SRP063665: http://www.ncbi.nlm.nih.gov/sra/SRP063665%5Baccn%5D, SRX1046562: http://www.ncbi.nlm.nih.gov/sra/SRX1046562%5Baccn%5D, and SRX1046561: http://www.ncbi.nlm.nih.gov/sra/SRX1046561%5Baccn%5D), and in the VB Genome Browser. FPs can be visualized with the VB Genome Browser at the following link:http://www.vectorbase.org/Aedes_aegypti/Location/View?db=core;r=supercont1.114:1965092-2050094;contigviewbottom=PRJNA294762_FAIREseq_Scheel_AaegL3=tiling. Access to the *A. aegypti* scaffolds reference v.4 to which these FPs were mapped is available at: https://www.vectorbase.org/downloadinfo/aedes-aegypti-liverpoolscaffoldsaaegl3fagz.

## Additional files

Additional file 1:
**Concordance of FAIRE-seq replicate experiments.** IDR analyses demonstrated the concordance of FAIRE-seq replicate experiments. Correspondence curves of matched peaks describing the function between the number of peaks in common and the number of significant peaks between replicates (A), within replicates (C), and within the merge of all three replicates (E) are shown. Correspondence curves of matched peaks describing the function between slope, representing the derivative, and illustrating the number of significant peaks between replicates (B), within replicates (D), and within the merge of all three replicates (F) are shown. (PDF 146 kb) Additional file 2:
**FP DNA Sequences.** The *A. aegypti* supercontig locations (start-end sites) for the entire FAIRE-seq data set are provided. (XLSX 3291 kb) Additional file 3:
**FPs immediately upstream of genes.** FPs within 1 kb upstream of the TSS of the indicated genes are noted. FP numbers correspond to the numbers assigned in Additional file 2. (XLSX 185 kb) Additional file 4:
**FPs mapping to 5’ UTRs.** FPs mapping to the 5’ UTRs of the indicated genes are noted. FP numbers correspond to the numbers assigned in Additional file 2. (XLSX 25 kb) Additional file 5:
**FPs mapping to 3’ UTRs.** FPs mapping to the 3’ UTRs of the indicated genes are noted. FP numbers correspond to the numbers assigned in Additional file 2. (XLSX 40 kb) Additional file 6:
**Intragenic FPs.** The number of intragenic FPs located within various genes is noted. FP numbers correspond to the numbers assigned in Additional file 2. (XLSX 243 kb) Additional file 7:
**FPs mapping to exons.** Genes with exonic FPs and the FP positions within exons are noted. FP numbers correspond to the numbers assigned in Additional file 2. (XLSX 14 kb) Additional file 8:
**FPs mapping to introns.** Genes with intronic FPs and FP positions within introns are noted. FP numbers correspond to the numbers assigned in Additional file 2. (XLSX 44 kb) Additional file 9:
**FPs mapping to intron-exon boundaries.** The intron-exon boundary locations of FPs in the indicated genes are noted. FP numbers correspond to the numbers assigned in Additional file 2. (XLSX 174 kb) Additional file 10:
**FPs associated with non-coding genes.** FPs within 1 kb upstream of the indicated non-coding genes are noted. The distance of the FP from the TSS of each gene is indicated. FP numbers correspond to the numbers assigned in Additional file 2. (XLSX 163 kb) Additional file 11:
**Moyo-S vs. Moyo-R SNPs mapping to FPs.** Moyo-S vs. Moyo-R SNPs at the indicated positions mapped to the FPs. The supercontig location of each SNP and corresponding FP ID numbers are indicated. FP numbers correspond to the numbers assigned in Additional file 2. (XLSX 181 kb) Additional file 12:
**Random sampling of FPs.** Bootstrap randomized sampling indicated that on average 25 SNPs were detectable in every 1000 FPs. (PDF 199 kb)

## Abbreviations

CRE: cis-regulatory element
CLOVER: cis-eLement OVER representation
DENV: dengue virus
EGFP: enhanced green fluorescent protein
FAIRE-seq: formaldehyde-assisted isolation of regulatory elements paired with DNA sequencing
FP: FAIRE peak
IDR: irreproducible discovery rate
IRES: internal ribosome entry site
LVP-IB12: Liverpool-IB12
MBE: Musashi-binding element
PAS: polyadenylation signal
SNP: single nucleotide polymorphism
TF: transcription factor
TSS: transcription start site
uORF: upstream open reading frame
UTR: untranslated region
VB: vectorbase
