# Supplementary figures and images for: High-throughput cis-regulatory element discovery in the vector mosquito Aedes aegypti
Source: BMC Genomics. 2016 May 10;17:341. doi: 10.1186/s12864-016-2468-x (PMC4862039; doi:10.1186/s12864-016-2468-x)

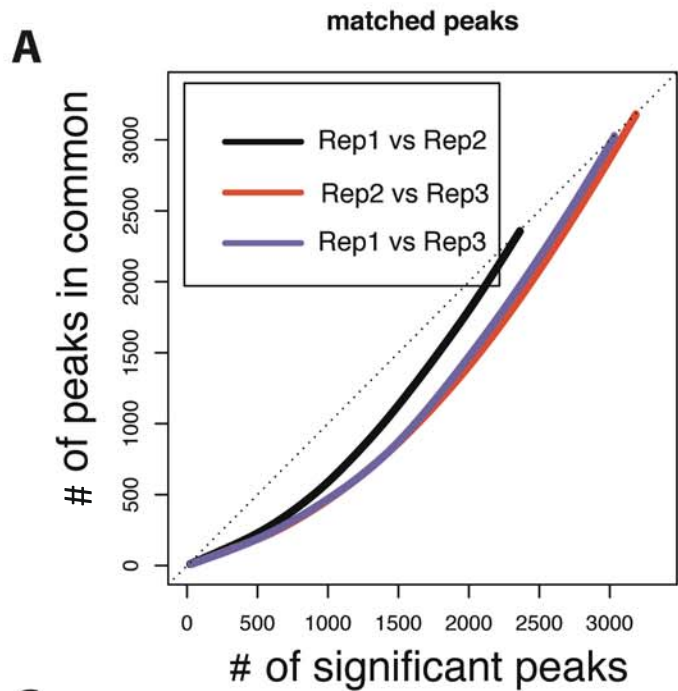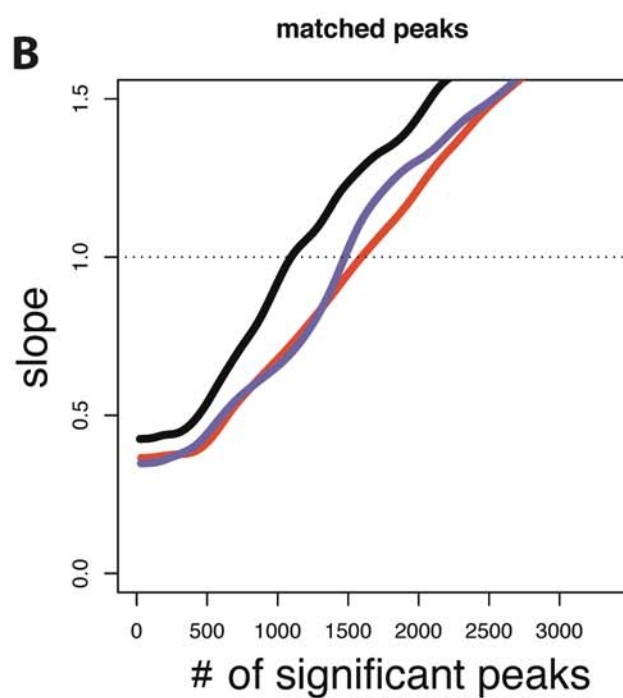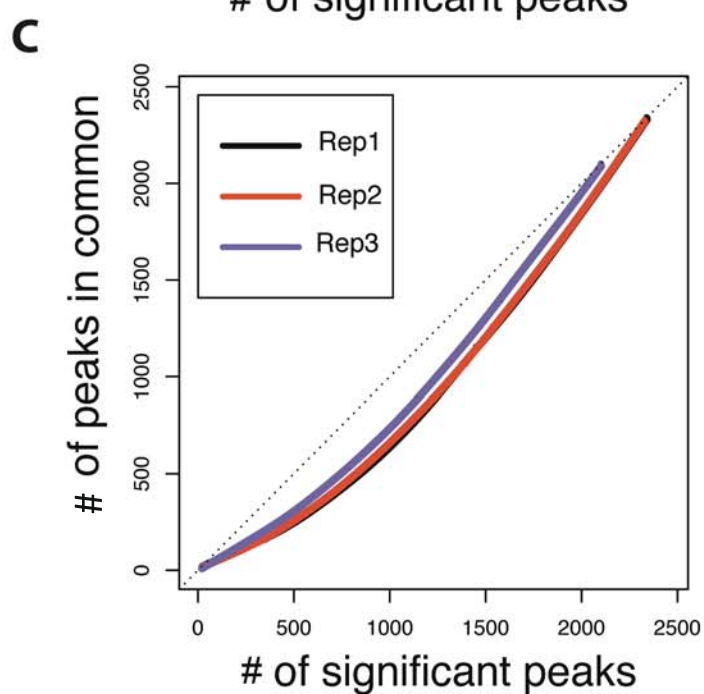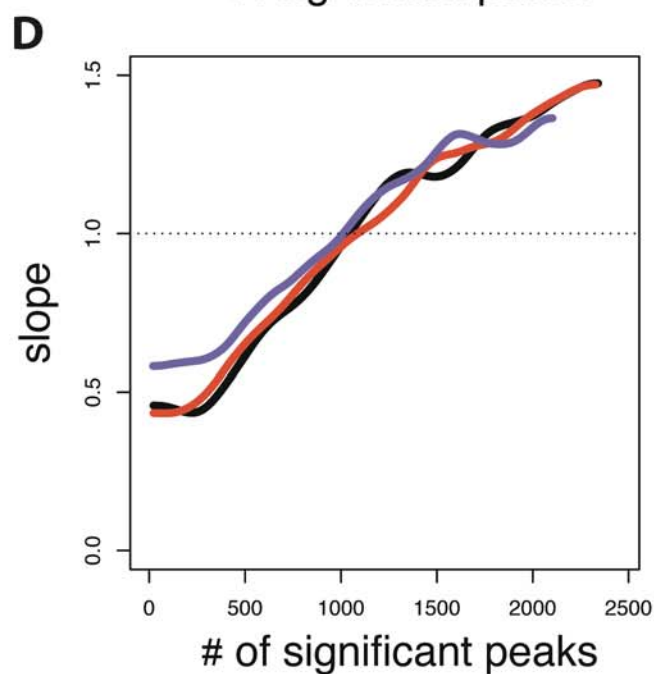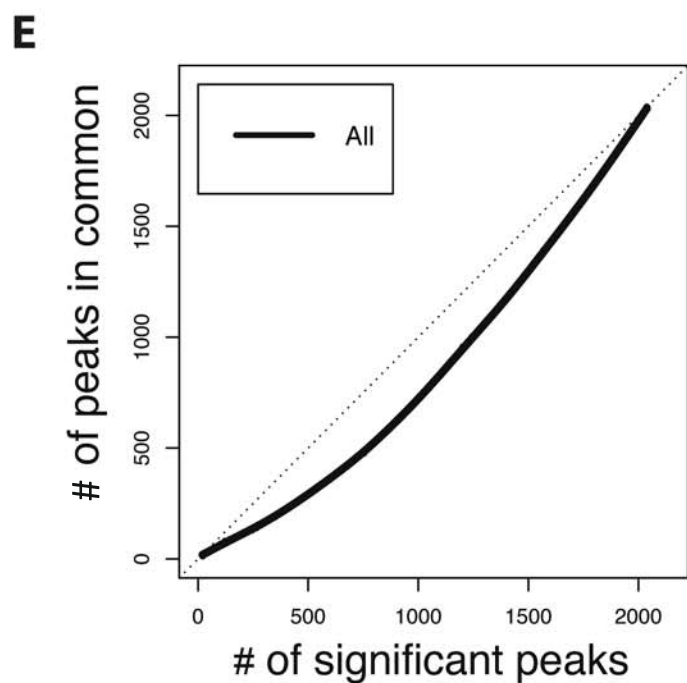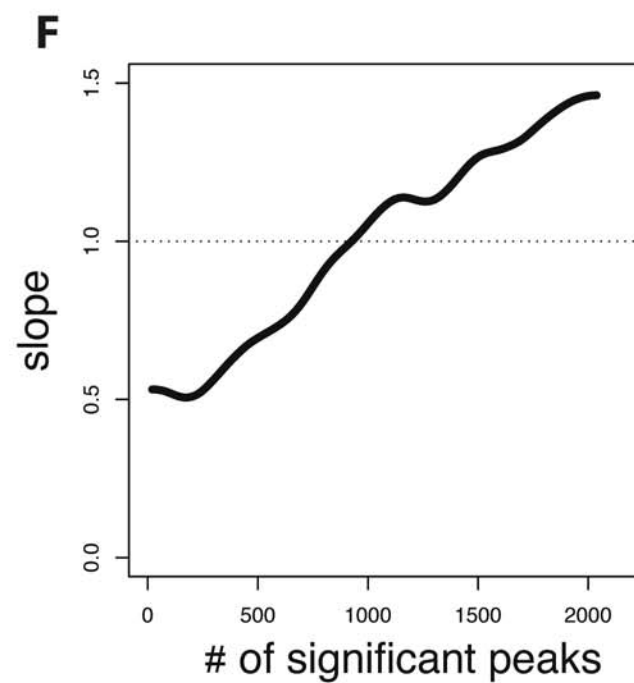

Supplement: Additional file 1: — Concordance of FAIRE-seq replicate experiments. IDR analyses demonstrated the concordance of FAIRE-seq replicate experiments. Correspondence curves of matched peaks describing the function between the number of peaks in common and the number of significant peaks between replicates (A), within replicates (C), and within the merge of all three replicates (E) are shown. Correspondence curves of matched peaks describing the function between slope, representing the derivative, and illustrating the number of significant peaks between replicates (B), within replicates (D), and within the merge of all three replicates (F) are shown. (PDF 146 kb) [file 12864_2016_2468_MOESM1_ESM.pdf]

## Random Sampling of FPs

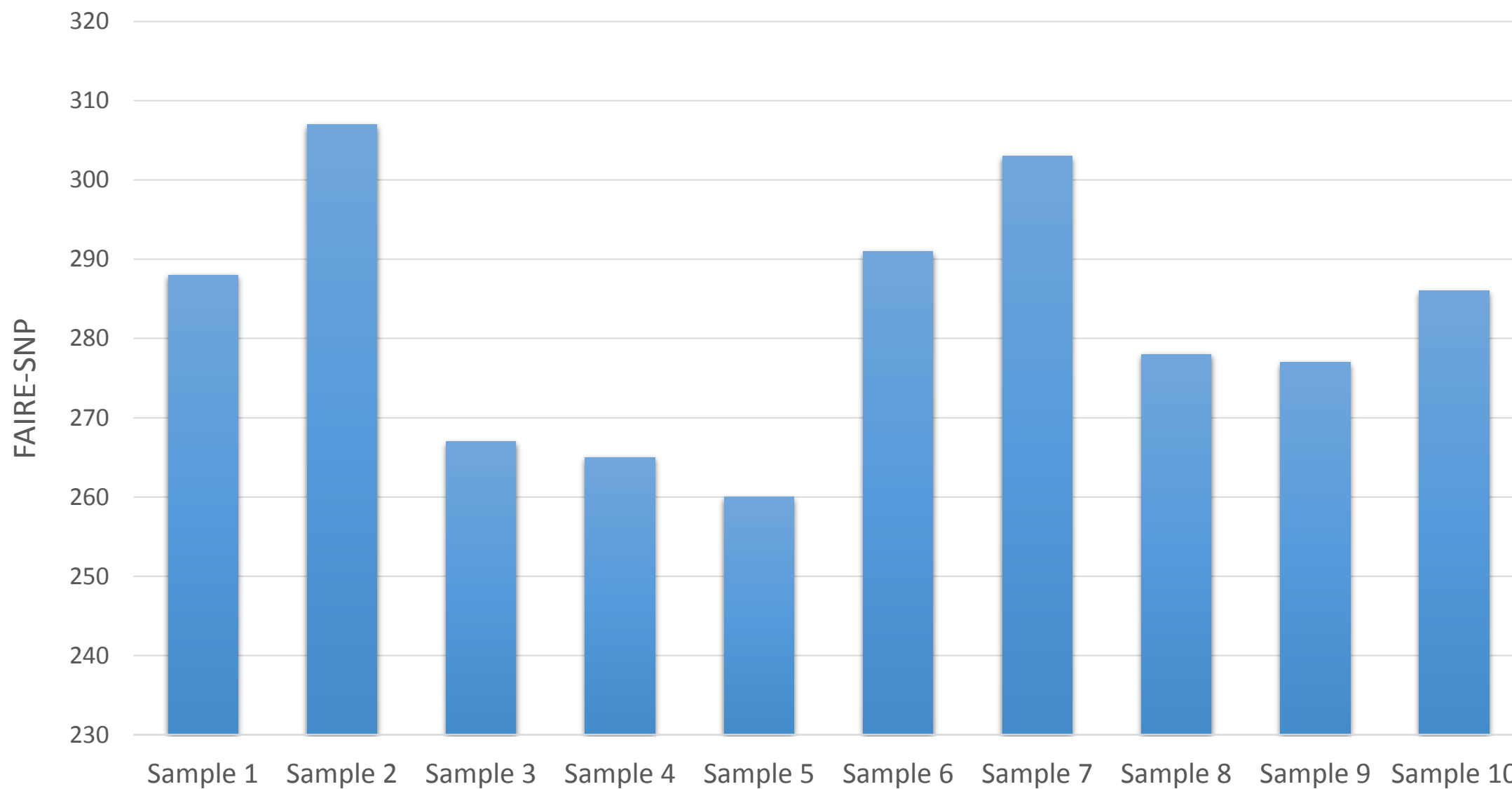

Supplement: Additional file 12: — Random sampling of FPs. Bootstrap randomized sampling indicated that on average 25 SNPs were detectable in every 1000 FPs. (PDF 199 kb) [file 12864_2016_2468_MOESM12_ESM.pdf]
